# The Influence of the Elemental Composition, Crystal Structure, and Grain Structure of the Ferro-Piezoceramics of Various Degrees of the Ferro-Hardness on the Stability of the Polarized State

**DOI:** 10.3390/ma15062118

**Published:** 2022-03-13

**Authors:** Konstantin Andryushin, Svetlana Dudkina, Sushrisangita Sahoo, Lidiya Shilkina, Vladimir Alyoshin, Ekaterina Triger, Inna Andryushina, Iliya Verbenko, Daniil Rudskiy, Angela Rudskaya, Larisa Reznichenko

**Affiliations:** 1 Research Institute of Physics, Southern Federal University, 344090 Rostov-on-Don, Russia; s.i.dudkina@yandex.ru (S.D.); sakhu@sfedu.ru (S.S.); lid-shilkina@yandex.ru (L.S.); vaalyoshin@sfedu.ru (V.A.); ilich001@yandex.ru (E.T.); futur6@mail.ru (I.A.); iaverbenko@sfedu.ru (I.V.); rudskiy@sfedu.ru (D.R.); lareznichenko@sfedu.ru (L.R.); 2 Faculty of Physics, Southern Federal University, 344090 Rostov-on-Don, Russia; agrudskaya@sfedu.ru

**Keywords:** ferro-piezoceramic, hot pressing, structure, microstructure, piezoelectric module, cycling

## Abstract

Ferro-piezoceramic materials (FPCM) with different degrees of ferrohardness were fabricated by double solid-phase synthesis followed by the sintering technique using hot pressing method. The X-ray studies carried out in a wide temperature range showed that with increasing temperature, each of the studied FPCM undergoes a series of phase transformations, accompanied by a change in the symmetry of the unit cell. In this case, near the phase transition to the nonpolar cubic phase, in each of the FPCM, the formation of a fuzzy symmetry region is observed, which is characterized by weak distortions and temperature–time instability of the crystal structure. The study of the piezoelectric modulus *d*_33_ in the quasi-static regime as a function of temperature made it possible to reveal the different nature of its behavior in materials of various degrees of ferrohardness. It was shown that the conservation of the state in ferrosoft materials above the Curie temperature is associated with the relaxation nature of the change in their properties, the existence of a region of fuzzy symmetry (noncubic phase) in them above the Curie temperature, and increased inertia of the system. The expediency of taking into account the presented results in the development of electromechanical converters based on FPCM of various degrees of ferrohardness, operated under temperature effects, including cyclic ones, was shown.

## 1. Introduction

In our present endeavor, a series of the highly efficient FPCM possessing the desired parameters for various applications has been developed at the Scientific Research Institute of Physics of the Southern Federal University [1]. These are the materials of nine groups, the ranges of the values of the main electrophysical characteristics and the areas of their use are presented in [2]. Most of them are based on the multicomponent systems of the complex oxides of the form Pb(Zr_1-x_Ti_x_)O_3_ (PZT)−$\sum_{n}{\left(\mathrm{Pb}B_{1-\alpha }^{\prime }B_{\alpha }^{\prime \prime }O_{3}\right)}_{n}$, where n = 1–4, with a wide variation of the physical parameters [3,4]. At the same time, the most demanded by the industry are the ferro-hard materials, FH, the medium ferro-hardness, MFH, and the soft ferroelectric materials, FS (ferrohardness is understood as the stability of the domain structure to the external influences [5]).

However, being extensively investigated from the material science point of view, these FPCM are still inadequately considered as the objects of physical consideration. At the same time, a significant obstacle on their path to physics was the fact that in these dirty, as it was believed, objects it was impossible to study physical phenomena and laws. However, the huge information accumulated to date indicates that the multicomponent FPCM of the above type are extremely interesting for the purely scientific research, since they exhibit the patterns conditioned by their complex hierarchical structure, which has no analogues in other solids [6,7,8,9,10,11].

In connection with the above, the purpose of the work was to establish the regularities of the influence of the elemental composition, crystal structure, and grain morphology of the ferroelectric piezoceramics of various degrees of the ferro-hardness on one of the most important characteristics—the stability of the polarized state under the influence of the temperature, modeling various real changes in the environment during the targeted operation of the FPCM in the form of the electromechanical converters.

## 2. Objects, Methods of Obtaining, and Studying the FPCM Samples

The objects of the study were the following FPCM:

- FH: PCR*-23, PCR-8; PCR-77M; PCR-78;

- MFH: PCR-87; PCR-6, PCR-86;

- FS: PCR-73; PCR-7M; PCR-7; PCR-66

^(^*^)^PCR—Rostov piezoceramics;

in which the B′—positions are occupied by Nb^5+^, Sb^5+^, W^6+^, and B″—Li^1+^, Mg^2+^, Ni^2+^, Zn^2+^, Mn^2+^, Cd^2+^. The main electrophysical characteristics of these FPCM at room temperature are given in [1,2].

For the experiments planned in this study, all of the above materials were prepared by the double solid-phase synthesis followed by the sintering technique using hot pressing method [12]. The technological regulations for each material: PCR-23, PCR-8, PCR-77M, PCR-78, PCR-87, PCR-6, PCR-86—T_synt.1,2_ = (1120 ÷ 1220) K, τ_synt.1,2_ = 5 h, T_sint._ = (1470 ÷ 1510) K; PCR-73, PCR-7M, PCR-7, PCR-66—T_synt.1,2_ = (1020 ÷ 1170) K, τ_synt.1,2_= 5 h, T_sint._ = (1370 ÷ 1420) K; for all compositions, the HP pressure P = 200 kg/cm^2^, and the isothermal holding time at the optimum sintering temperature τ = 40 min.

Manufacturing of the measuring samples included two technological steps: the mechanical processing and electrode deposition. The samples were made in the form of the disks of ∅ 10 mm × 1 mm. The surface treatment was performed with the diamond tool (TegraPol-11, Struers A/S, Ballerup, Denmark) according to the sixth class of accuracy. The electrodes were applied by double firing a silver-containing paste (LLC “Rostechnohim”, Rostov-on-Don, Russia) at the temperature of (1070 ± 20) K for 0.5 h. For the microstructural and X-ray studies, one sample was specially prepared from a series of the samples of each composition, the flat surface of which was polished to grade 13.

The formation of the polarized state was performed by the method of the hot polarization, in which an electric field was applied to the samples at a high temperature. The rational method of the polarization was chosen; such samples were loaded into a chamber with PES-5 polyethylene siloxane liquid (LLC “Rostechnohim”, Rostov-on-Don, Russia) at 330 K. During (0.5 ÷ 1.5) h (depending on the composition), the temperature was gradually raised to 410 K, accompanied by an increase in the generated field from 0 to (5 ÷ 7) kV/mm. Under these conditions, the samples were exposed for (20 ÷ 25) min. They were then cooled under the field to (330 ÷ 360) K for at least 0.5 h. The measured (ρ_meas_) density of the samples was determined by the method of the hydrostatic weighing in octane (LLC “Rostechnohim”, Rostov-on-Don, Russia).

The X-ray studies were performed by the powder diffraction using DRON-3 and ADP diffractometers (JSC Innovative Center “BUREVESTNIK”, St. Petersburg, Russia) (Fe_Kα_-radiation; Mn-filter; Fe_Kβ_-radiation; Bragg-Brentano focusing scheme). The crushed ceramic objects were investigated, which made it possible to exclude the influence of the surface effects, stresses, and textures arising in the process of obtaining the ceramics. The calculation of the structural parameters was performed according to the standard methods [13]. The measurement errors of the structural parameters had the following values: linear Δa = Δb = Δc = ±(0.002 ÷ 0.004) Å; angular Δβ = 3′; volume ΔV = ±0.05 Å^3^ (ΔV/V∙100% = 0.07%, T—the tetragonal phase, Rh—the rombohedral phase, Pcs—the pseudocubic (the fuzzy symmetry phase), RFS—the region of the fuzzy symmetry. The uniform strain parameter, δ, was calculated using the formulas δ = 2/3(c/a − 1) (for the T-phase).

The calculation of the X-ray density (ρ_x-ray_) was performed according to the formula: ρ_x-ray_ = 1.66∙M/V, where M—the weight of the formula unit in grams, V—the volume of the perovskite cell in Å. The relative density (ρ_rel_) was calculated using the formula (ρ_meas_/ρ_x-ray_)∙100%. 

The study of the polycrystalline structure (the microstructure) of the ferroelectric materials was performed in the reflected light on an optical microscope Neophot 21 (Carl Zeiss, Oberkochen, Germany). The samples were preliminarily ground on the fine-grained abrasive paper. Then, finer grinding was performed on the free abrasive having a particle size of D ≤ 5 μm in the presence of an aqueous medium. Further, the samples were also polished in an aqueous medium using the Cr_2_O_3_ powder with a particle size of 0.1 µm to 0.2 µm. The processing quality at the last two stages was monitored using a microscope.

The visualization of the intercrystalline boundaries of the ferroelectric ceramics was performed by the method of the chemical etching with a 5% aqueous solution of concentrated nitric acid (LLC “Rostechnohim”, Rostov-on-Don, Russia) with the addition of 15 drops of concentrated hydrofluoric acid (LLC “Rostechnohim”, Rostov-on-Don, Russia) per 200 mL of the etchant. The etching time, as well as the etchant concentration, was selected for each material according to the nature of the realized picture. To analyze the microstructure of the FPCM, the optical micrographs and approximated images of the areas of the polished surface were used. In this case, the approximation means the digital processing of the original images of the surface areas. The black-and-white photographs of the surface of the objects obtained with an optical microscope were subjected to the computer processing, as a result of which the extraneous noise was excluded and a clean mesh of the ceramic grain boundaries was revealed.

**Figure 1:**
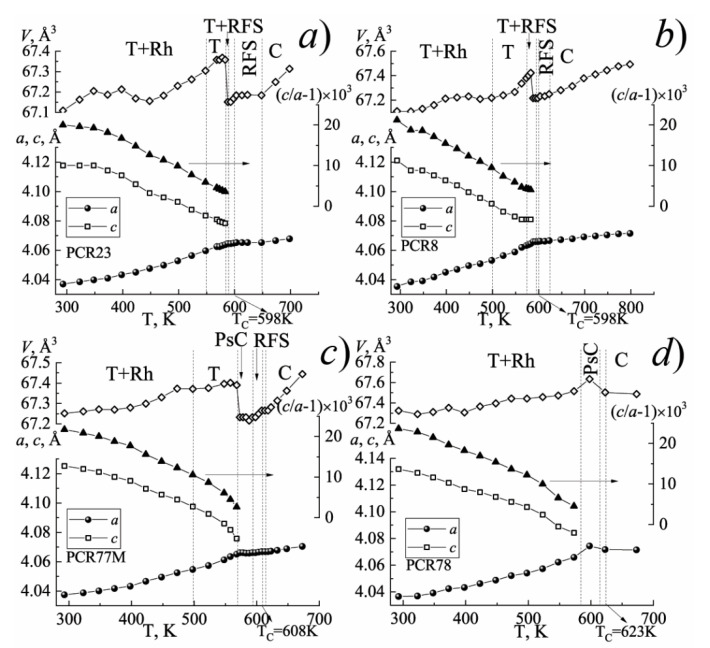
The dependences of the structural parameters of the FH-materials on the temperature: (**a**) PCR23; (**b**) PCR8; (**c**) PCR77M; (**d**) PCR78. (The vertical dashed lines indicate the phase boundaries).

High-temperature studies of the relative permittivity of unpolarized samples, (*ε*/*ε*_0_), were carried out on an LCR-meter E-7-20 instrument (JCS “MNIPI”, Minsk, Belarus) at frequencies of 25 Hz–1 MHz in the temperature range (300–920) K. The temperature was controlled using a Varta 703I temperature controller (St. Petersburg, Russia) and a chromel-aluminum thermocouple. The temperature determination error is ± 0.5 K. The Curie temperatures were determined from the totality of X-ray data, i.e., change in the symmetry of the unit cell, as well as the peak *ε*/*ε*_0_ at a frequency of 1 kHz. Comparative analysis of X-ray data and dielectric spectroscopy (for example, see Figure 1) showed the practical coincidence of the formation of the phase transition to the cubic phase, which indicates the reliability of the data presented.

The piezoelectric modulus *d*_33_ in the quasi-static mode as a function of temperature was determined on a sample in the form of a disk using a specially designed stand based on a *d*_33_ m YE2730A. Temperature control was also carried out using a Varta 703I temperature controller and a chromel-aluminum thermocouple. The temperature determination error is ± 0.5 K.

The studies of *d*_33_ = *f*(T) were performed by three methods. Method 1: the sample was gradually heated once until the complete loss of the piezoactivity with a step ΔT = 5 K. Method 2: the sample was heated to the temperature of the partial annealing, passing the values of *T*_0*i*_ with a step ΔT = 5 K, while the temperature of the partial annealing was calculated by the formula: $T_{oi}=T_{oi}+\sum_{i=2}^{4}\frac{T_{k}-T_{o1}}{n}$ (T_c_ is the Curie temperature, *n* is the number of the cycles); after reaching the temperature *T_0i_*, the sample was also stepwise cooled to room temperature with the fixation *d*_33_; the heating-cooling cycles followed one after another without the interruption, and on the last cycle the sample was heated to the temperature of the complete loss of the piezoactivity. Method 3: the sample was heated to the temperature ≈ 353 K below T_C_ with a step ΔT = 10 K; after reaching the maximum temperature, T_m_, the sample was also stepwise cooled to room temperature with the fixation *d*_33_; the heating-cooling cycles followed one after the other without the interruption.

## 3. Results

**Figure 2:**
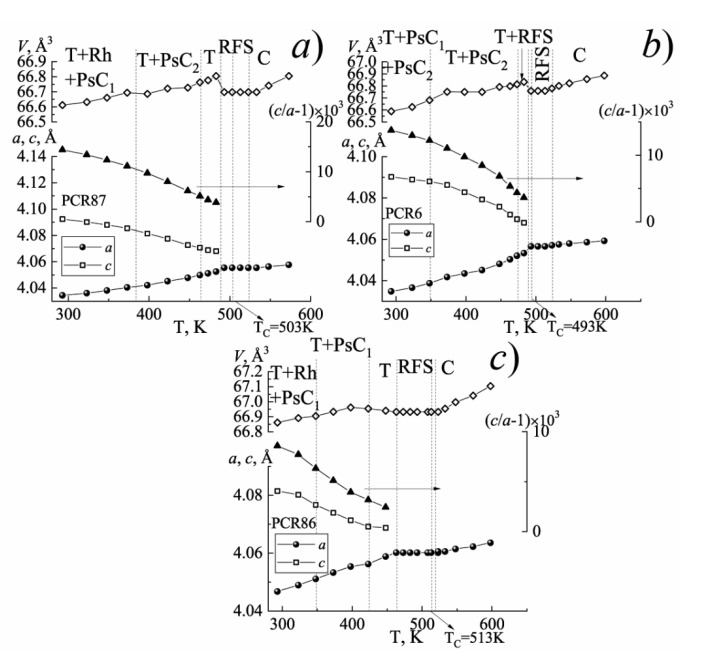
The dependences of the structural parameters of the MFH-materials on the temperature: (**a**) PCR87; (**b**) PCR6; (**c**) PCR86. (The vertical dashed lines indicate the phase boundaries).

**Figure 3:**
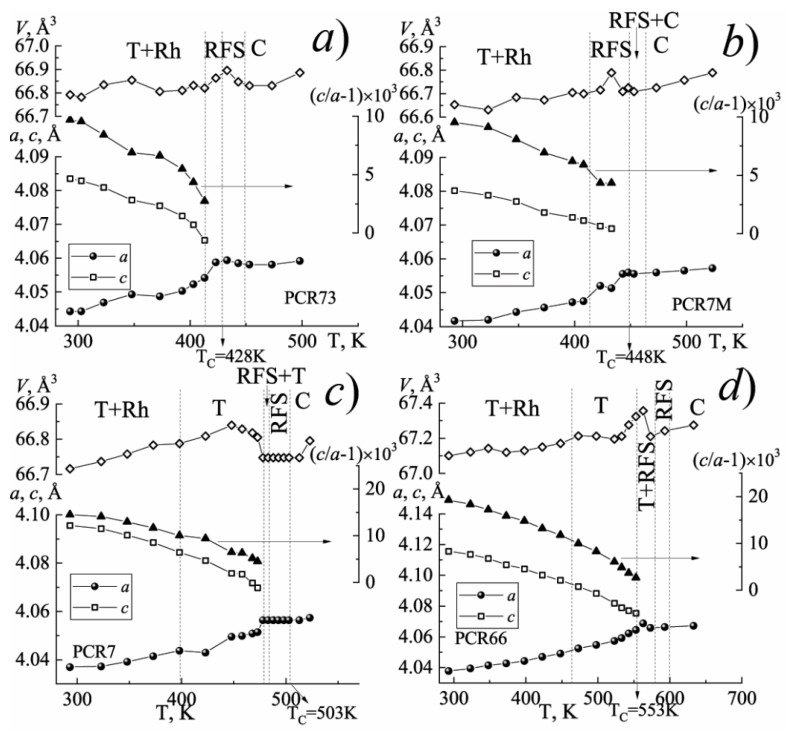
The dependences of the structural parameters of the FS-materials on the temperature: (**a**) PCR73; (**b**) PCR7M; (**c**) PCR7; (**d**) PCR66. (The vertical dashed lines indicate the phase boundaries).

Figure 1, Figure 2 and Figure 3 show the structural characteristics of the studied SS in the range (300 ÷ 720) K.

The analysis of Figure 1 has shown that with an increase in the temperature, each of the investigated FPCM undergoes a number of the phase transformations accompanied by a change in the symmetry of the unit cell. In this case, near the phase transition to the nonpolar cubic phase, in each of the FPCM, the formation of the region of the fuzzy symmetry (RFS) is observed, which is characterized by the weak distortions and temperature–time instability of the crystal structure. 

The position and length (in the temperature) of such RFS depend on the elemental composition of the FPCM and, as a consequence, the ferro-hardness of the materials. As the FPCM “hardens” the width of the RFS narrows.

**Figure 4:**
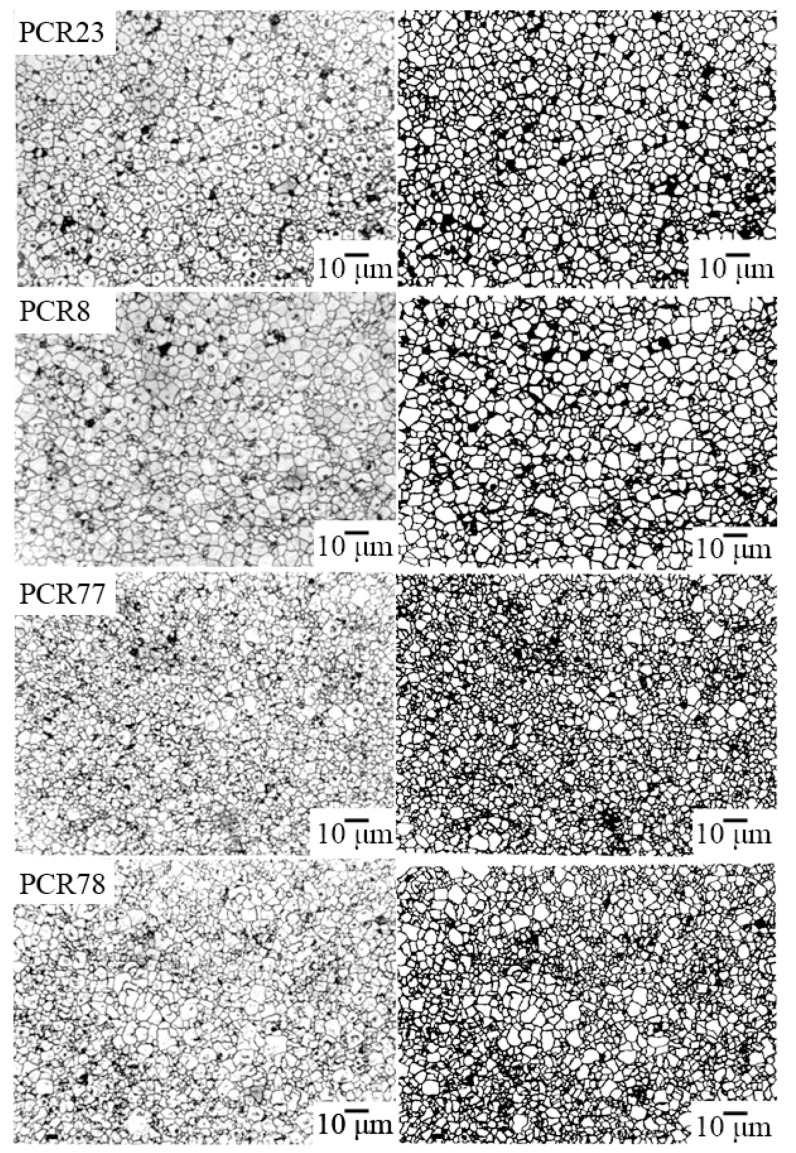
The optical micrographs (on the **left**) and the approximated images (on the **right**) of the areas of the polished surface of the FH-materials.

**Figure 5:**
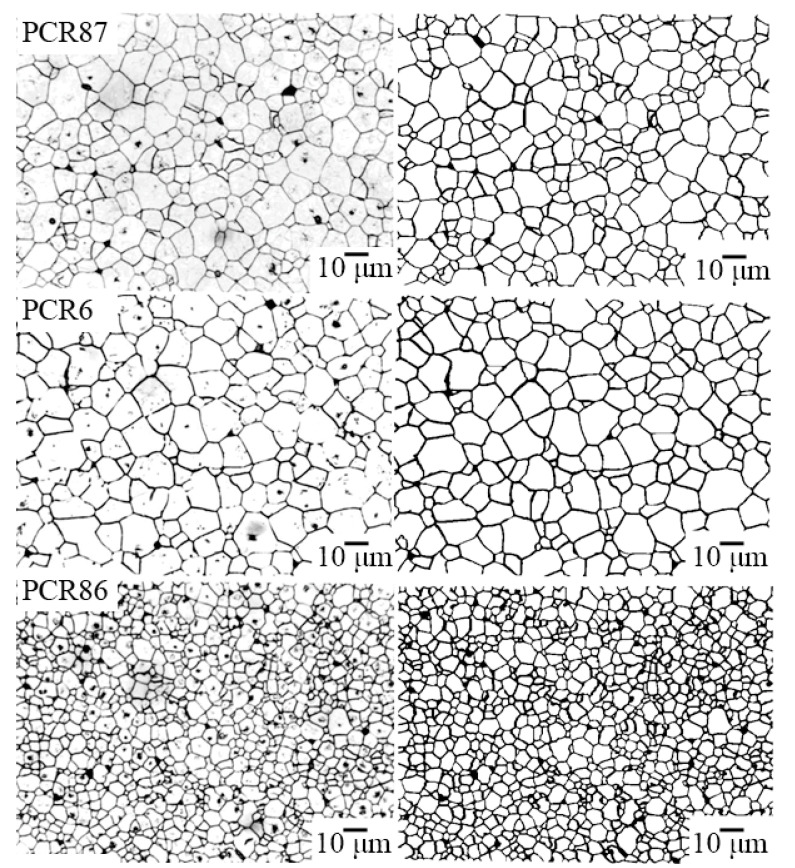
The optical micrographs (on the **left**) and the approximated images (on the **right**) of the areas of the polished surface of the MFH-materials.

**Figure 6:**
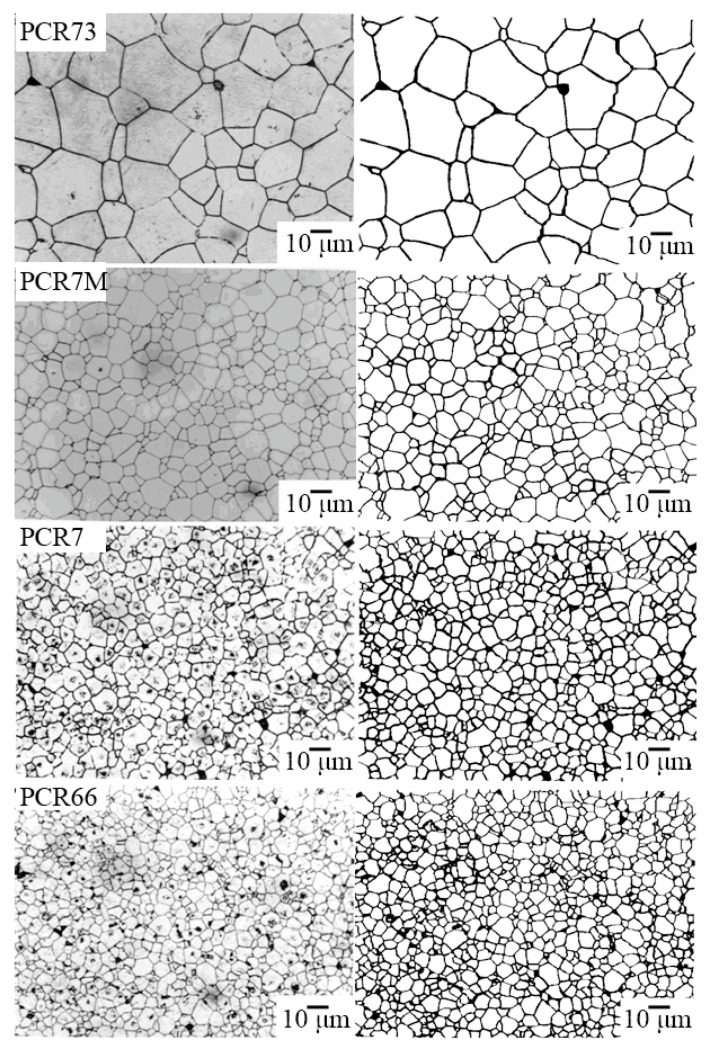
The optical micrographs (on the **left**) and the approximated images (on the **right**) of the areas of the polished surface of the FS-materials.

Figure 4, Figure 5 and Figure 6 show the photomicrographs and approximated images of the areas of the polished surface of the investigated FPCM. Common to all grain landscapes is the formation of the densely packed structures of the varying degrees of homogeneity with the crystallites of the irregular shape, framed by the thin curved boundaries. The grain structure of the FS-materials is the most inhomogeneous. To a lesser extent, this is typical for the MFH media, and in the FH objects against the background of an extremely fine-grained structure, this effect manifests itself in the form of the well-defined regions with larger crystallites. 

Such an inhomogeneity of the microstructure of the studied ceramics is undoubtedly a consequence of the peculiarity of their crystalline structure—two-phase, associated with the localization of all the analyzed materials inside or in the vicinity of the morphotropic region (MR) of the corresponding solid solution (SS) systems.

**Figure 7:**
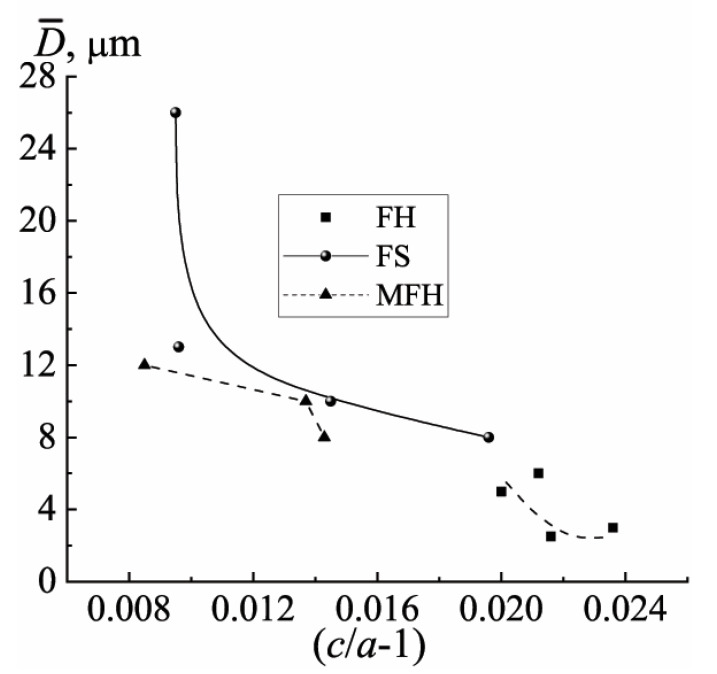
The dependences of the average grain size on the deformation of the T-cell in the materials of different ferro-hardness.

In all the groups of the materials, an inverse dependence of the crystallite size (*D*) on the degree of the deformation of the crystalline (tetragonal) cell of the FPCM ((*c*/*a*) − 1) is observed (Figure 7). This is typical for all the types of the ferropiezoceramics [14] and is explained by an increase in the internal stresses in the FPCM with an increase in the spontaneous deformation. It is these stresses that inhibit the growth of the crystallites due to the implementation of the unfavorable conditions for the diffusion processes and mass transfer during the recrystallization sintering.

**Figure 8:**
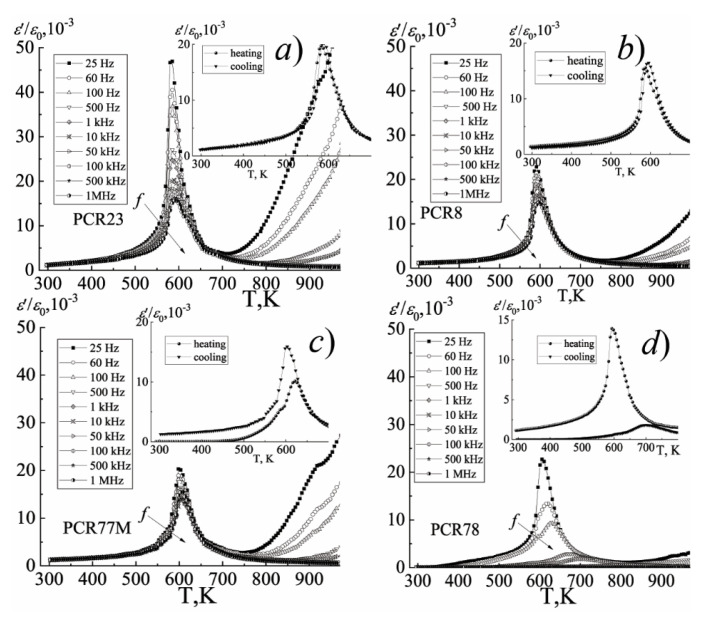
The dependences of *ε*/*ε*_0_ on the temperature of the FH-materials: (**a**) PCR23; (**b**) PCR8; (**c**) PCR77M; (**d**) PCR78 (the cooling mode, *f* = (25 − 10^6^) Hz). The inset shows the dependence of *ε*/*ε_0_*(T) (the heating-cooling mode, *f* = 10^4^ Hz). The temperature measurement step is Δ*T* = 5 K.

**Figure 9:**
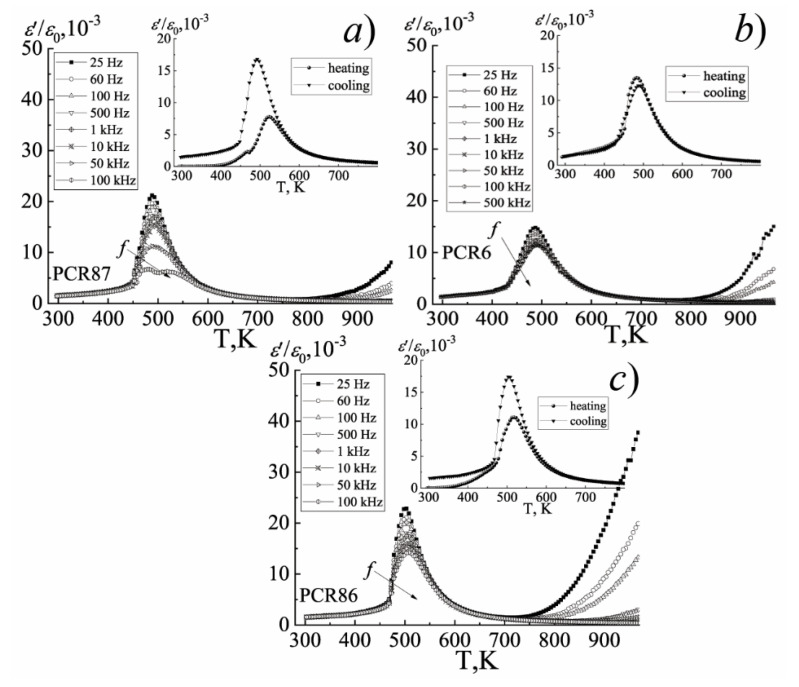
The dependences of *ε*/*ε*_0_ on the temperature of the MFH-materials: (**a**) PCR87; (**b**) PCR6; (**c**) PCR86 (the cooling mode, *f* = (25 − 10^6^) Hz). The inset shows the dependence of *ε*/*ε_0_* (T) (the heating-cooling mode, *f* = 10^4^ Hz). The temperature measurement step is Δ*T* = 5 K.

**Figure 10:**
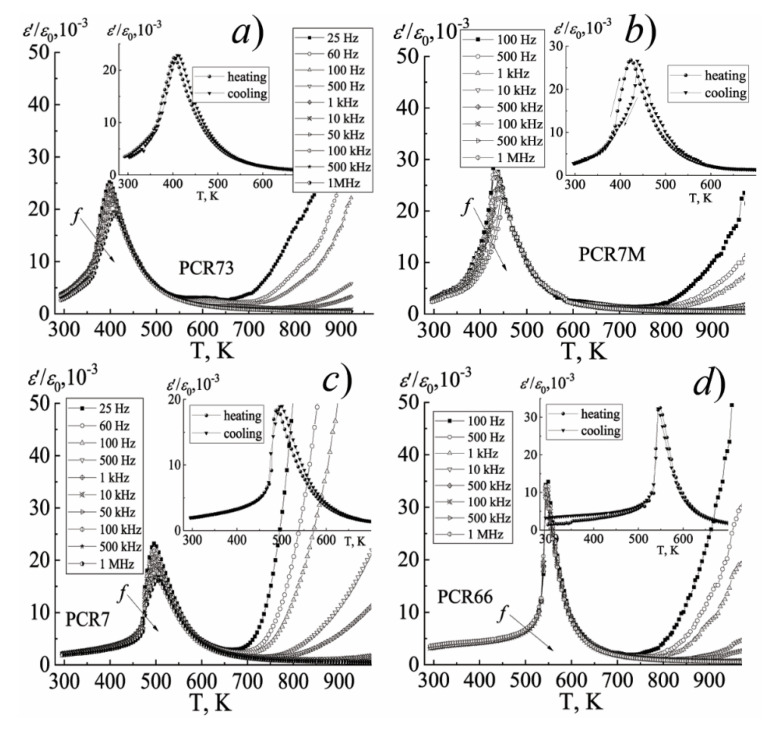
The dependences of *ε*/*ε*_0_ on the temperature of the FS-materials: (**a**) PCR73; (**b**) PCR7M; (**c**) PCR7; (**d**) PCR66 (the cooling mode, *f* = (25 − 10^6^) Hz). The inset shows the dependence of *ε*/*ε_0_*(T) (the heating-cooling mode, *f* = 10^4^ Hz). The temperature measurement step is Δ*T* = 5 K.

The difference in the nature of the behavior of the dielectric constant of the FPCM when the temperature and frequency of the measuring field change is clearly visible on Figure 8, Figure 9 and Figure 10. 

Thus, in the group of the FS-materials, the diffuse maxima of *ε*/*ε*_0_ are observed, their decrease and shift to the region of higher temperatures with the increasing frequency, which is characteristic of the ferroelectric relaxors. In the group of the MFH- and FH-materials, the position of the maximum of *ε*/*ε*_0_ does not depend on the frequency of the measuring field, which is typical for classical ferroelectrics. All this is undoubtedly related to the specificity of the elemental composition of the FPCM, based, in the case of the FS- materials, on the ferroelectric compounds of the relaxor type PbNb_2/3_Mg_1/3_O_3_, PbNb_2/3_Zn_1/3_O_3_, PbNb_2/3_Ni_1/3_O_3_ [15,16,17,18,19,20], and in the case of the FH- and MFH-materials—on the Mn-containing compositions such as PbNb_2/3_Mn_1/3_O_3_, PbW_1/3_Mn_1/2_O_3_, PbSb_2/3_Mn_1/3_O_3,_ etc. [4]. The hardening effect of the latter is provided by the crystal-chemical characteristics of Mn. Firstly, by a higher degree of the covalence of the Mn-O bonds due to the higher electronegativity of Mn^3+^, Mn^4+^ (always present in the composition of the FPCM due to the redox processes during their synthesis and sintering), which contributes to an increase in the spontaneous deformation and, as a consequence, to ferro-hardness of the materials. Secondly, by the manifestation of the Jahn–Teller effect by Mn^3+^, which makes an additional contribution to the overall distortion of the unit cell. Thirdly, by the small size of the Mn^3+^ and Mn^4+^ ions, which facilitates their occupation of the vacant positions, which stabilizes and toughens the structure. Finally, by the possibility of the formation of the liquid phases of the eutectic origin due to the low melting temperatures of the manganese oxides, which cements the grains and reduces the mobility of the domain structures [4]. 

Figure 8, Figure 9 and Figure 10 show the dependences of *ε*/*ε*_0_ on the temperature of the materials under study.

A sharp increase in *ε*/*ε*_0_ in the paraelectric region (in the vicinity of (670–820)K) may be a consequence of the redox processes associated with variable valences of Nb^(5+^^↔4+)^ and Ti^(4+^^↔3+)^ [21,22,23], and as a consequence, the appearance of the oxygen vacancies, which form the anion-deficient nonstoichiometry. These vacancies, weakly related to the structure of the material, are the sources of the conductivity and make an additional contribution to the formation of the dielectric properties of the solid solutions.

**Figure 11:**
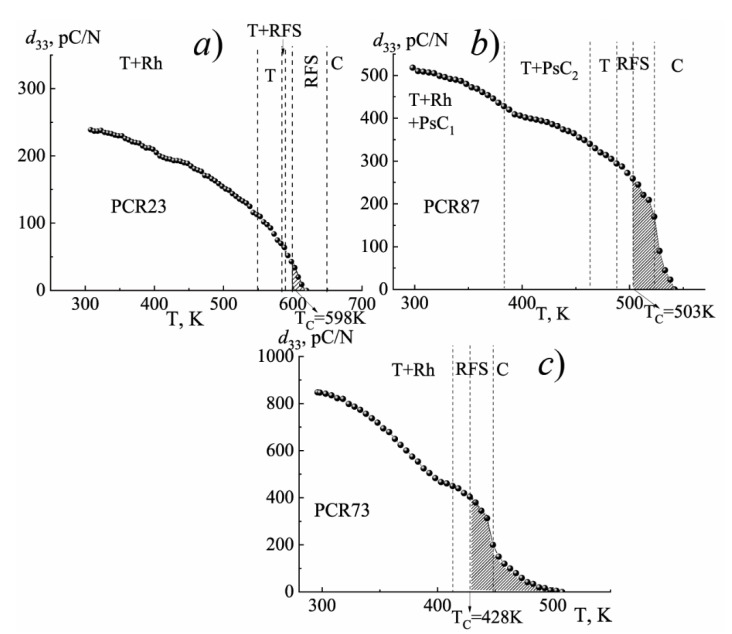
The changes in the piezomodule *d*_33_ of the FH (**a**), MFH (**b**), FS (**c**) materials in the course of a conventional (single) increase in the temperature (method №1). (The vertical dashed lines indicate the phase boundaries. The region of the conservation of the piezoactive state above the Curie temperature, T_C_, is shaded).

**Figure 12:**
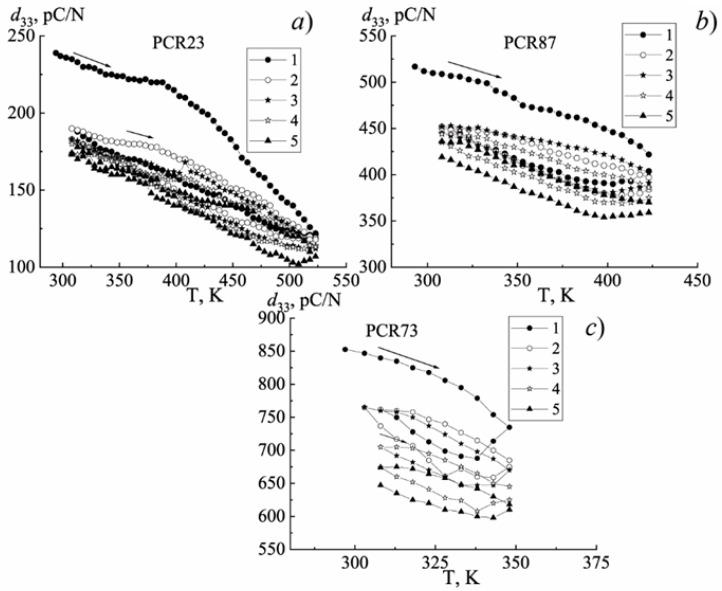
The dependence of the piezomodule *d*_33_ on the temperature of the FH (**a**), MFH (**b**), FS (**c**) materials in the process of a multiple increase in the temperature (method №2). (The numbers indicate the heating–cooling cycles).

**Figure 13:**
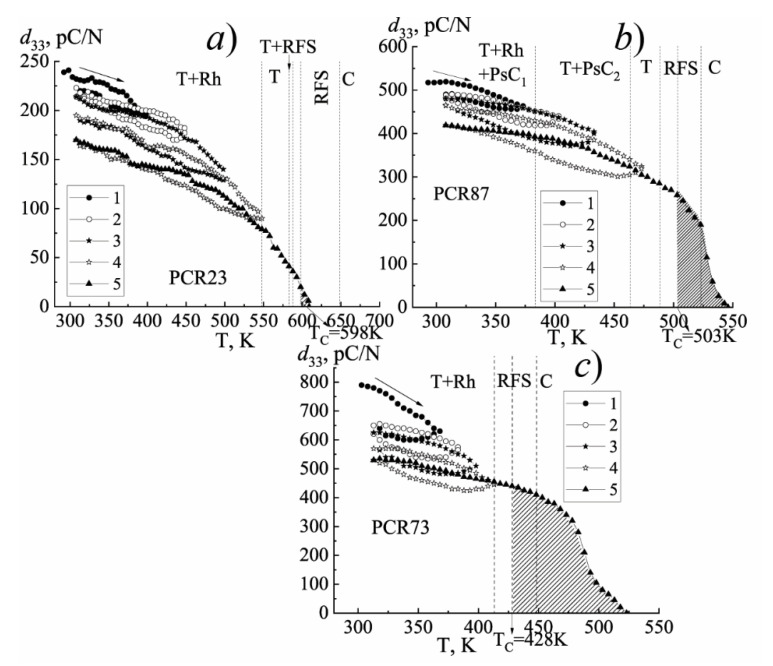
The change of the piezomodule *d*_33_ of the FH (**a**), MFH (**b**), FS (**c**) materials in the process of increasing the temperature by method №3. (The numbers indicate the heating-cooling cycles).

Figure 11, Figure 12 and Figure 13 illustrate the behavior of the piezomodule *d*_33_ of the materials under study in the process of changing the temperature by various cycling methods.

**Figure A1:**
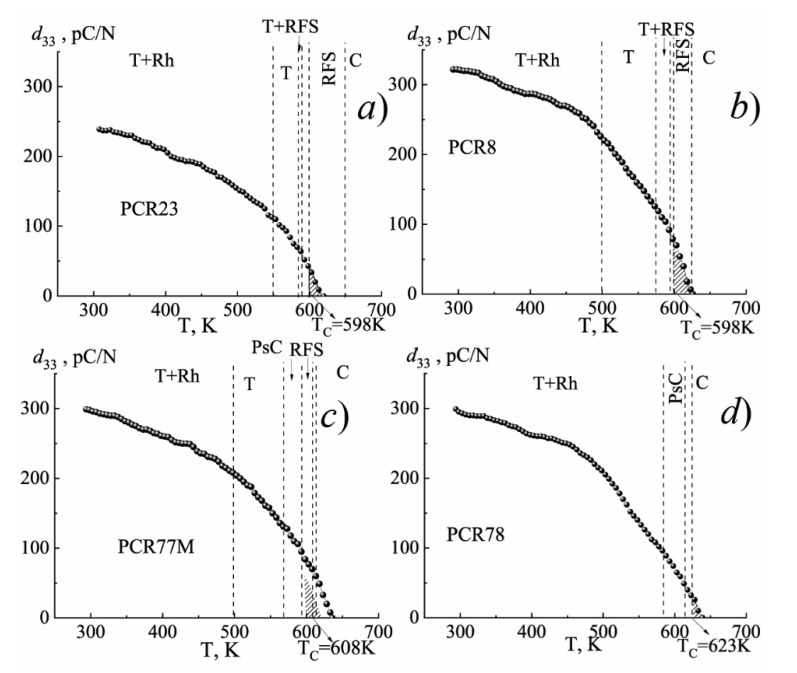
The changes in the piezomodule *d*_33_ of the FH-materials in the course of a conventional (single) increase in the temperature (method 1): (**a**) PCR23; (**b**) PCR8; (**c**) PCR77M; (**d**) PCR78. (The vertical dashed lines indicate the phase boundaries. The region of the conservation of the piezoactive state above the Curie temperature, T_C_, is shaded).

**Figure A2:**
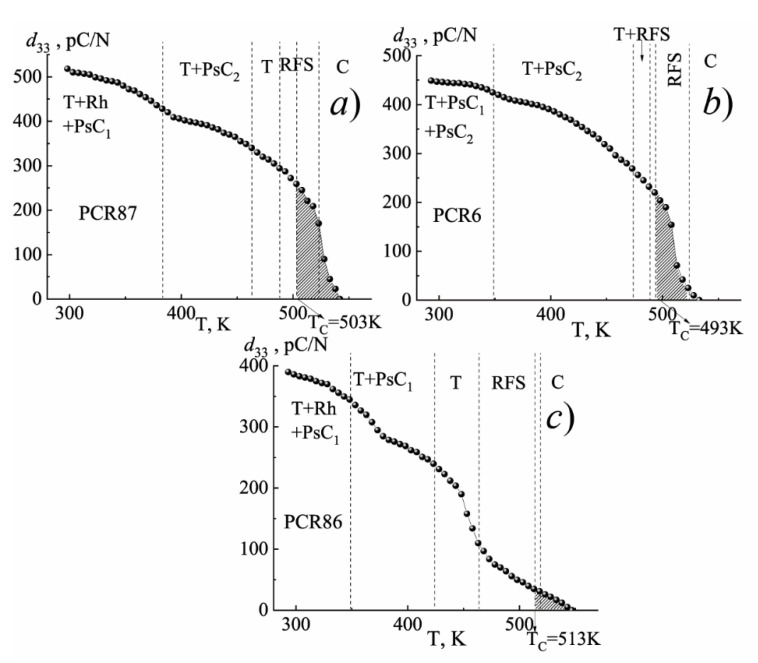
The changes in the piezomodule *d*_33_ of the MFH-materials in the course of a conventional (single) increase in the temperature (method №1): (**a**) PCR87; (**b**) PCR6; (**c**) PCR86. (The vertical dashed lines indicate the phase boundaries. The region of the conservation of the piezoactive state above the Curie temperature, T_C_, is shaded).

**Figure A3:**
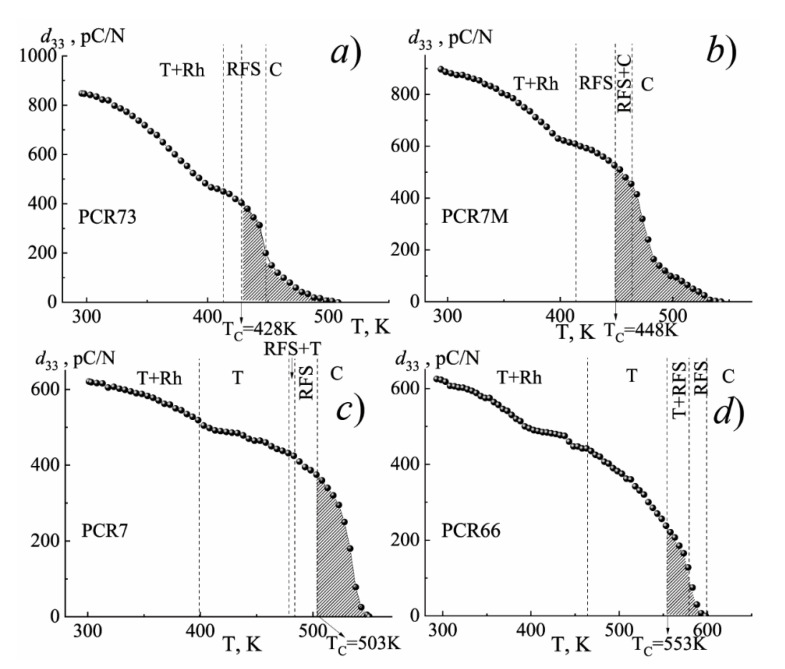
The changes in the piezomodule *d*_33_ of the FS-materials in the course of a conventional (single) increase in the temperature (method №1): (**a**) PCR73; (**b**) PCR7M; (**c**) PCR7; (**d**) PCR66. (The vertical dashed lines indicate the phase boundaries. The region of the conservation of the piezoactive state above the Curie temperature, T_C_, is shaded).

As can be seen from the graphs shown in Figure 11 (method № 1) (for all studied SS belonging to FH-materials—photos are presented in Appendix A. Figure A1, Figure A2 and Figure A3), with increasing the temperature *d*_33_ decreases at different rates in the regions differing in the symmetry of the unit cell. In this case, the changes in the slope of the *d*_33_(T) dependences correspond to the phase boundaries. The highest rate of the change in *d*_33_ is possessed by the FS-materials due to the greater mobility of the domain structure, which readily responds to the external influences, including the temperature. With the hardening of the materials, the rate of the change of *d*_33_ decreases, becoming minimal in the FH media.

**Figure A4:**
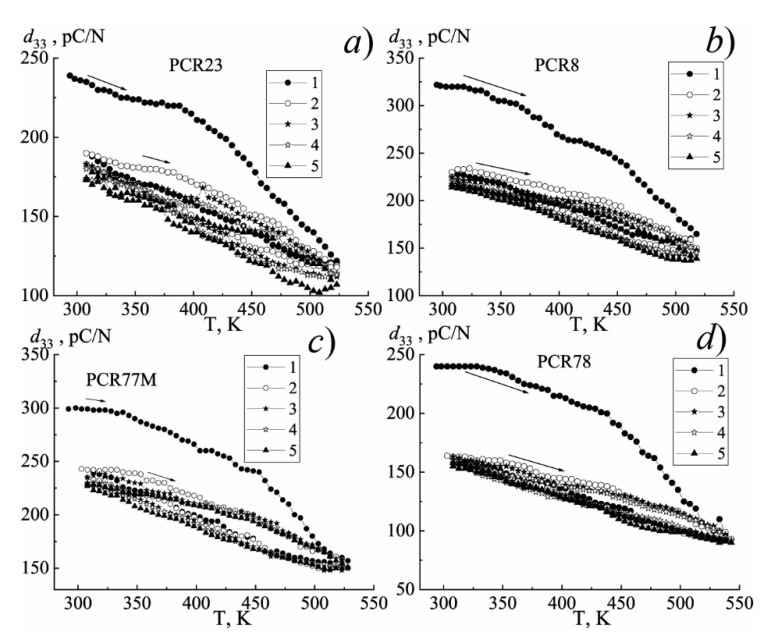
The dependence of the piezomodule *d*_33_ on the temperature of the FH-materials in the process of a multiple increase in the temperature (method №2): (**a**) PCR23; (**b**) PCR8; (**c**) PCR77M; (**d**) PCR78. (The numbers indicate the “heating–cooling” cycles).

**Figure A5:**
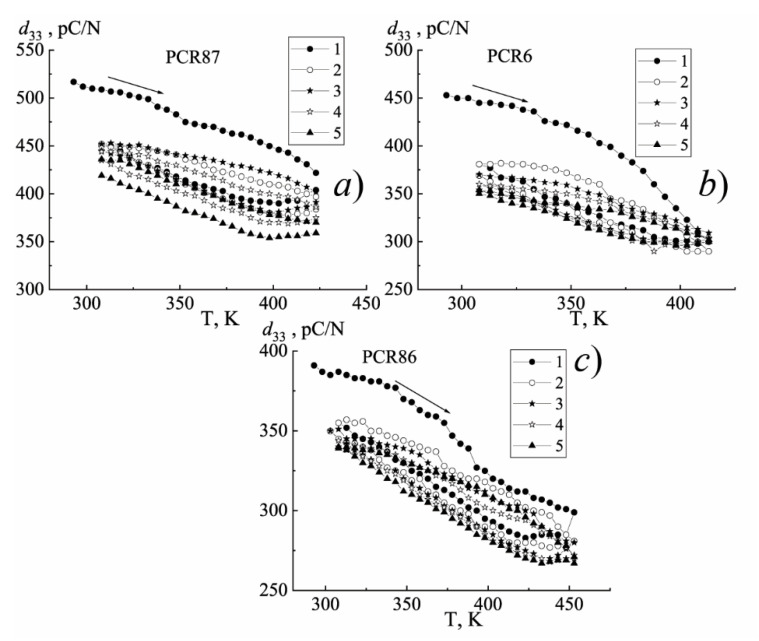
The dependence of the piezomodule *d*_33_ on the temperature of the MFH-materials in the process of a multiple increase in the temperature (method №2): (**a**) PCR87; (**b**) PCR6; (**c**) PCR86. (The numbers indicate the “heating–cooling” cycles).

**Figure A6:**
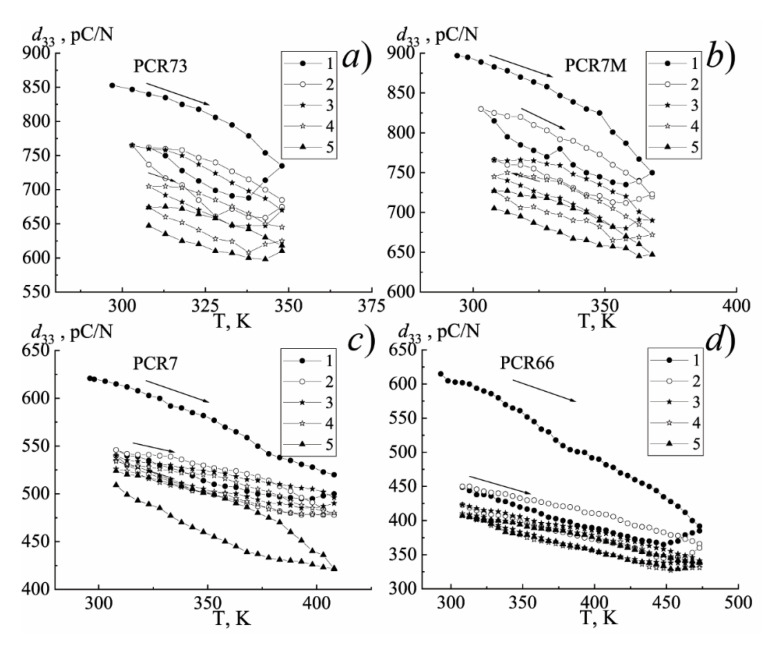
The dependence of the piezomodule *d*_33_ on the temperature of the lubricating FH-materials in the process of a multiple increase in the temperature (method №2): (**a**) PCR73; (**b**) PCR7M; (**c**) PCR7; (**d**) PCR66. (The numbers indicate “heating–cooling” cycles).

When the temperature is cycled by method №2, *d*_33_ decreases during heating, and with subsequent cooling, it increases (Figure 12) (for all studied SS belonging to MFH-materials—photos are presented in Appendix A. Figure A4, Figure A5 and Figure A6). Moreover, the heating-cooling cycles are located under each other and are practically parallel to each other, which indicates the same rate of the change in *d*_33_. Only during the last heating, when passing through the Curie temperature, T_C_, *d*_33_ begins to decrease faster. With the hardening of the materials, the distance between the cycles and the rates of descending of *d*_33_ decreases, as in method №1.

**Figure A7:**
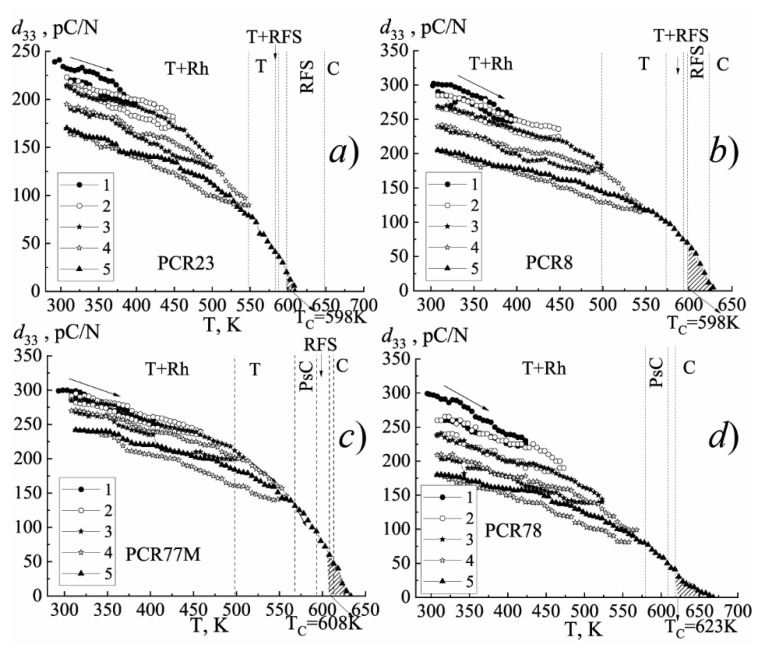
The change of the piezomodule *d*_33_ of the FH-materials in the process of increasing the temperature by method №3: (**a**) PCR23; (**b**) PCR8; (**c**) PCR77M; (**d**) PCR78. (The numbers indicate the “heating–cooling” cycles).

**Figure A8:**
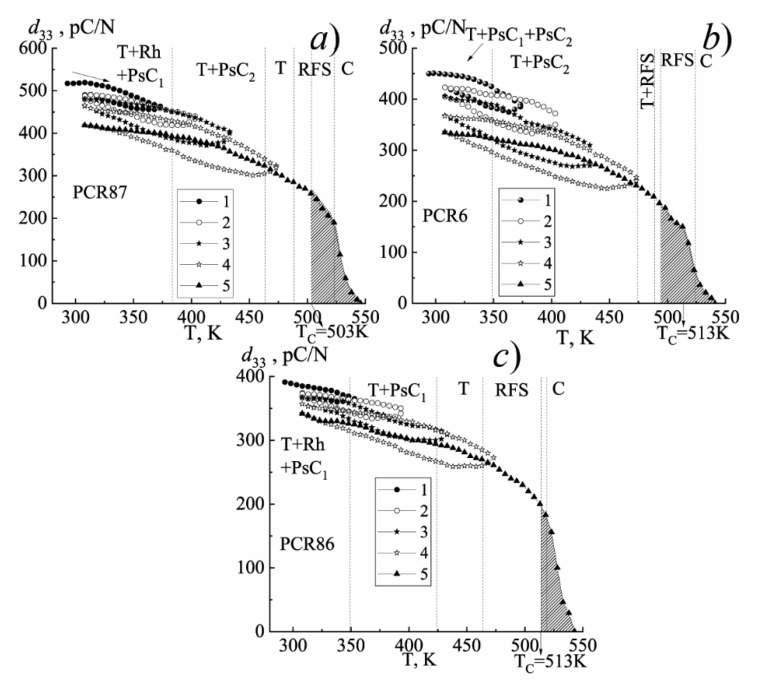
The change of the piezomodule *d*_33_ of the MFH-materials in the process of increasing the temperature by method №3: (**a**) PCR87; (**b**) PCR6; (**c**) PCR86. (The numbers indicate the “heating–cooling” cycles).

**Figure A9:**
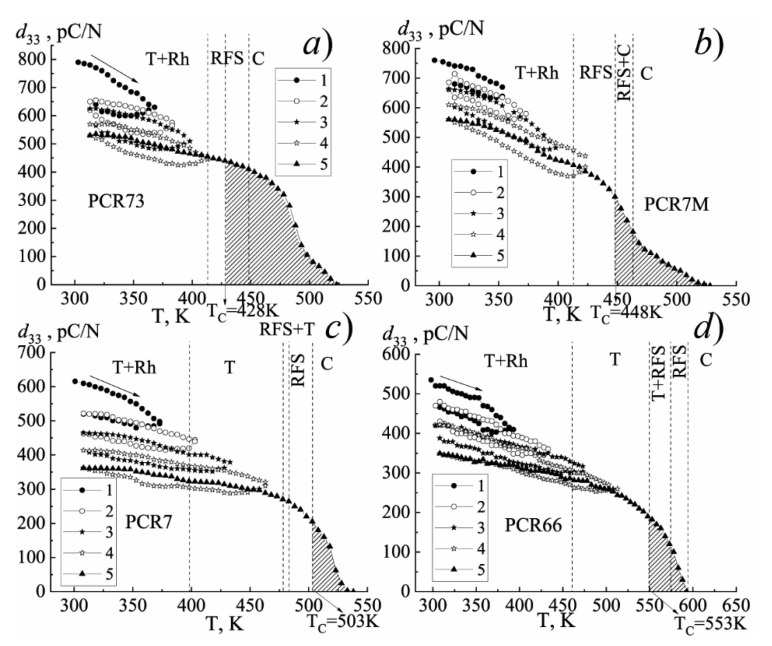
The change of the piezomodule *d*_33_ of the FH-materials in the process of increasing the temperature by method №3: (**a**) PCR73; (**b**) PCR7M; (**c**) PCR7; (**d**) PCR66. (The numbers indicate the “heating–cooling” cycles).

Comparing the graphs in Figure 13 (method №3) (for all studied SS belonging to MFH-materials—photos are presented in Appendix A. Figure A7, Figure A8 and Figure A9), a decrease in *d*_33_ with the temperature up to the heating temperature can be noticed. When cooling down on the first cycle, *d*_33_ restores its value by 60–70%. The same is observed during the subsequent cycling. In this case, the *d*_33_ values in the heating and cooling modes are lower than the corresponding values of the first cycle. However, starting from the third cycle, the situation changes: the decrease of the piezomodule slows down. In the last cycles, the difference between the *d*_33_ values is very small and the cycles overlap.

It should be noted that the piezoactive state still exists above T_C_ for FPCM as indicated by the shaded region (Figure 13c). We also observed that the lower the ferrohardness of the material was, the wider this region in the paraelectric phase (Figure 13). The above observation can be attributed to the relaxor nature of the FS materials and the existence of noncubic phases in the vicinity of T_C_. 

## 4. Conclusions

1. The different behavior of the piezomodule *d*_33_ in the materials of various degrees of ferro-hardness under the influence of the temperature in three schemes has been revealed. The highest rate of the change in the piezomodule, regardless of the measurement modes, is observed in the FS-materials due to the mobility of the domain structure, which readily responds to the external influences, including temperature.
2. The observed conservation of the piezoactive state in the FS-materials above the Curie temperature is associated with the relaxation nature of the change in their properties, the existence of a region of the fuzzy symmetry (noncubic phase) in them above the Curie temperature, and the increased inertia of the system.
3. The interpretation of the obtained experimental results is given taking into account the influence of the elemental composition, crystal structure, and grain structure of the ferropiezoceramics of various degrees of the ferro-hardness on the stability of the polarized state.

It is advisable to take into account the presented results when developing electromechanical converters based on the FPCM of various degrees of ferro-hardness, operated under the influence of the temperature, including cycling.

## Data Availability

Not applicable.
